# Comparative Phenotypic and Genotypic Analysis of Swiss and Finnish *Listeria monocytogenes* Isolates with Respect to Benzalkonium Chloride Resistance

**DOI:** 10.3389/fmicb.2017.00397

**Published:** 2017-03-23

**Authors:** Anja B. Meier, Claudia Guldimann, Annukka Markkula, Anna Pöntinen, Hannu Korkeala, Taurai Tasara

**Affiliations:** ^1^Institute for Food Safety and Hygiene, Vetsuisse Faculty University of ZurichZurich, Switzerland; ^2^Department of Food Hygiene and Environmental Health, Faculty of Veterinary Medicine, University of HelsinkiHelsinki, Finland

**Keywords:** *Listeria monocytogenes*, benzalkonium chloride, bcrABC, qacH, emrE, MLST

## Abstract

Reduced susceptibility of *Listeria monocytogenes* to benzalkonium chloride (BC), a quaternary ammonium compound widely used in food processing and hospital environments, is a growing public health and food safety concern. The minimal inhibitory concentration of BC on 392 *L. monocytogenes* strains from Switzerland (CH) and Finland (FIN) was determined. Within this strain collection, benzalkonium chloride resistance was observed in 12.3% (24/195) of Swiss and 10.6% (21/197) of Finnish strains. In both countries, the highest prevalence of BC-resistant strains (CH: 29.4%; FIN: 38.9%) was detected among serotype 1/2c strains. Based on PCR analysis, genes coding for the *qacH* efflux pump system were detected for most of the BC-resistant strains (CH: 62.5%; FIN: 52.4%). Some Swiss BC-resistant strains harbored genes coding for the *bcrABC* (16.7%) efflux pump system, while one Finnish BC-resistant strain harbored the *emrE* gene previously only described among BC-resistant *L. monocytogenes* strains from Canada. Interestingly, a subset of BC-resistant strains (CH: 5/24, 20.8%; FIN: 9/21, 42.8%) lacked genes for efflux pumps currently known to confer BC resistance in *L. monocytogenes*. BC resistance analysis in presence of reserpine showed that the resistance was completely or partially efflux pump dependent in 10 out of the 14 strains lacking the known BC resistance genes. Sequence types 155 and ST403 were over-representated among these strains suggesting that these strains might share similar but yet unknown mechanisms of BC resistance.

## Introduction

*Listeria monocytogenes*, the causative agent of listeriosis in humans and animals, represents a major foodborne pathogen with serious impacts on public health and the food industry (de Valk et al., 2005; Popovic et al., 2014; Crim et al., 2015). Listeriosis mainly, but not exclusively affects neonates, elderly people, pregnant women, and immunosuppressed individuals and may cause gastroenteritis, sepsis, central nervous system infections, and abortion in pregnant women (Maertens de et al., 2014). Listeriosis, even though relatively rare, occurs worldwide (Maertens de et al., 2014) and is of major public health concern due to the high case-fatality rate in human clinical cases (15–30 deaths/100 cases; Crim et al., 2015; de Valk et al., 2005; Popovic et al., 2014). The ubiquitous nature of *L. monocytogenes* and its ability to grow at refrigeration temperatures (Walker et al., 1990) and to tolerate very low pH (reviewed in Smith et al., 2013) and high salt concentrations (Bergholz et al., 2010), increases the risk of foodborne outbreaks from strains that achieve high concentrations on products with a long shelf life and subsequently cause human infections upon consumption. Strains of *L. monocytogenes* can persist in niches within food processing facilities for years, representing a serious food safety issue (reviewed in Ferreira et al., 2014). As a preventive measure, quaternary ammonium compounds (QACs) such as benzalkonium chloride (BC), are widely used for cleaning and disinfection of food processing environments (McDonnell and Russell, 1999; Mereghetti et al., 2000). Quaternary ammonium compounds function by disrupting cell membranes of bacteria, subsequently leading to leakage of the cytosol, and degradation of proteins as well as nucleic acids (McDonnell and Russell, 1999). *L. monocytogenes* strains with low susceptibility to BC have been regularly isolated from foods and food processing environments. (Mereghetti et al., 2000; Romanova et al., 2002; Mullapudi et al., 2008; Fox et al., 2011)., Benzalkonium chloride resistant (BC^r^) strains have been isolated from human listeriosis infections (Elhanafi et al., 2010), and there is evidence of cross-protection against other antimicrobials including gentamicin and ciprofloxacin (Rakic-Martinez et al., 2011). In light of this, the presence of BC^r^ strains in food and food processing environments is concerning.

The known molecular mechanisms of BC resistance are due to the activity of efflux pump systems encoded through the *brcABC* (Elhanafi et al., 2010), *qacH* on the Tn6188 transposon (Müller et al., 2013), and *emrE* (Gilmoure et al., 2010; Kovacevic et al., 2015) genes that can be acquired by horizontal gene transfer leading to BC resistance in *L. monocytogene*s. There is limited knowledge of the prevalence of BC resistance among Swiss *L. monocytogenes* strains; a recent study found a prevalence of 18% among 142 Swiss strains isolated from food and the food processing environment (Ebner et al., 2015). Virtually no information is available on the QAC resistance profiles in strains from Finland. The aim of this study was to analyze and compare a large collection consisting of *L. monocytogenes* strains from Switzerland and Finland for BC susceptibility and the presence of known genes that convey resistance to QAC. The strain collection included isolates originating from samples taken along the whole length of the food production chain, ranging from the farm environment (silage, feces of farm animals, birds, and veterinary clinical cases) to food production facilities and various foods to human clinical cases.

## Materials and methods

### Bacterial strains

The 392 *L. monocytogenes* strains used in this study were collected between 1999 and 2013 in Switzerland and Finland (Supplementary Table 1). The Swiss strains (*n* = 195) were collected at the Swiss National Reference Centre for Enteropathogenic Bacteria and Listeria (NENT). This strain collection differed from that recently described in the study by Ebner et al. (2015). Finnish strains (*n* = 197) were collected through the Department of Food Hygiene and Environmental Health of the Faculty of Veterinary Medicine at the University of Helsinki. Unlike the Swiss strain collection, the Finnish strain collection lacked human clinical isolates. The origin of the strains was summarized as: dairy (strains isolated from dairy products), meat (strains isolated from carcasses and raw meat products), fish (strains isolated from raw fish), ready to eat (RTE) food (strains isolated from RTE seafood, salad, sausage, ham, maize products), vegetables (strains isolated from raw vegetables), food animals (strains isolated from cows, goats, pigs, and sheep), birds (strains isolated from the feces of wild birds), food production environments (FPE; strains isolated from meat, RTE and dairy production environments), human (strains isolated from human listeriosis cases), others (strains isolated from quorn, rice, silage). Bacteria were stored at −80°C in brain heart infusion (BHI; Oxoid, Pratteln, Switzerland) broth plus 20% glycerol (Sigma-Aldrich, Buchs, Switzerland).

### Strain serotyping and BC susceptibility testing

Strain serotypes were assigned by the slide agglutination test using the commercial set of Listeria O-factor and H-factor antisera from Denka Seiken (Pharma Consulting, Burgdorf, Switzerland) according to the manufacturer's instructions. Susceptibility to BC was tested using the previously described agar dilution method (Elhanafi et al., 2010). Strains were plated on blood agar plates (Difco, Columbia blood agar base, 5% sheep blood, Oxoid) and incubated for 18 h at 37°C. Single colonies were picked from each plate on the next day and suspended in 100 μl of Mueller Hinton broth (MHB; Oxoid, Pratteln, Switzerland). Five microliters of the suspensions were spotted in technical duplicates on Mueller Hinton Agar (MHA; Oxoid) plates supplemented with 2% defibrinated sheep blood (Oxoid) and various BC concentrations (0, 2.5, 5, 7.5, 10, 15, 20, 25, and 30 μg ml^−1^; Sigma-Aldrich). Benzalkonium chloride minimal inhibitory concentrations (MICs) were read after incubating the plates at 37°C for 48 h. Spots could exhibit either no growth, growth of individual colonies, or confluent growth over the full area of the spot. Strains were considered resistant to a given concentration of BC if the spots showed confluent growth, and the MIC was defined as the lowest BC concentration preventing confluent growth of the spotted bacteria. Strains were considered BC^r^ if they exhibited confluent growth at or above 20 μg ml^−1^. This cutoff was defined after the following considerations: we first determined the lowest BC concentration that killed >50% of all strains (10 μg ml^−1^). Based on (Langsrud et al. (2003); Xu et al. (2014), we then defined resistance at a MIC that was double this concentration (20 μg ml^−1^). Minimal inhibitory concentrations are indicated as >30 μg ml^−1^ for strains that exhibited confluent growth at 25 and 30 μg ml^−1^ BC.

### Impact of efflux pump inhibition with reserpine on BC susceptibility

To assess the contribution of efflux pump activity in BC^r^ strains, the BC MICs of such strains were also determined on MHB agar plates containing various BC concentrations (0, 2.5, 5, 7.5, 10, 15, 20, 25, and 30 μg ml^−1^; Sigma-Aldrich) and supplemented with the efflux pump inhibitor reserpine (20 μg ml^−1^; Sigma-Aldrich; Romanova et al., 2006). The BC resistance of the *L. monocytogenes* strains was classified as not efflux dependent (no effect of reserpine on the BC MIC), partially efflux pump dependent (addition of reserpine resulted in a decrease of the BC MIC of <10 μg ml^−1^), or fully efflux pump dependent (addition of reserpine resulted in a decrease of the BC MIC of ≥10 μg ml^−1^).

### Genetic analysis of all BC^r^ strains

The DNA templates were extracted from *L. monocytogenes* strains that were grown overnight in BHI broth (37°C and 125 rpm), using the DNeasy blood and tissue kit (Qiagen). Genotyping by multilocus sequence typing (MLST) was performed as previously described (Ragon et al., 2008). Polymerase chain reactions (PCRs) to amplify seven housekeeping genes were performed using the HotStartTaq Master Mix (Qiagen) and 50 ng of genomic DNA template from each analyzed strain. The PCR products were sequenced at Microsynth (Balgach). The MLST types and genetic lineages were assigned using the *L. monocytogenes* MLST database website (http://bigsdb.web.pasteur.fr). The PCR analysis for the presence of *bcrABC*, Tn*6188*, and *emrE* genes was performed as previously described using primers shown in Table 1. The *emrE* primers were designed based on *L. monocytogenes* strain sequence 05–5578 described by Gilmoure et al. (2010). The R56 and R159 *L. monocytogenes* strains (Ebner et al., 2015) were used as *bcrABC* and Tn*6188* positive controls, respectively whereas the LR39-1 strain (Kovacevic et al., 2012) was used as a positive control for *emrE*.

**Table 1:** **Primers used in this study**.

**Primer**	**Genetic target**	**Sequence (5′-3′)**	**References**
p1	*bcrABC*	CAT TAG AAG CAG TCG CAA AGC A	Elhanafi et al., 2010
p2		GTT TTC GTG TCA GCA GAT CTT TGA	
radC fwd	*Tn6188*	CTT GCC AAT GAT AAT ATC ATC	Müller et al., 2013
radC rev		GTG GTC TGA ATG CTC CAT CG	
EmrE fw	*emrE*	GAC CAA CAC CAC CTA AGT	This study
EmrE rv		GTC TGA TGG ACT TAC AAA GCT	

### Statistical analysis

Statistical analysis was performed using the JMP program (Version 11.0.0, SAS Institute Inc., NC, USA). Fisher's exact test was used in a series of individual pairwise comparisons using 2 × 2 tables to compare proportions of BC^r^ and BC^s^ within the serotypes and the sources per country. *P* < 0.05 were considered to be statistically significant.

## Results

### Serotypes and origins of swiss and finnish *L. monocytogenes* strains

A panel of 195 Swiss (CH) and 197 Finnish (FIN) *L. monocytogenes* strains that were isolated from diverse sources including foods, food production environments, food animals, wild birds, and human listeriosis cases was serotyped. Table 2 presents an overview of the distribution of these strains based on serotypes and isolation sources. Strains examined from these two countries belonged to serotypes 1/2a (CH: 105/195, 53.8%, and FIN: 134/197, 68.0%), 4b (CH: 47/195, 24.1%; FIN: 23/197, 11.7%), 1/2b (CH: 26/195, 13.3%; FIN: 18/197, 9.1%), 1/2c (CH: 17/195, 8.7%; FIN: 18/197, 9.1%), and 3a (CH: 1/195, 0.5%; FIN: 4/197, 2.0%). In terms of isolation sources, the Swiss strains came from human listeriosis (80/195, 41.0%), meat (59/195, 30.3%), RTE food (17/195; 8.7%), dairy (14/195, 7.2%), FPE (17/195, 8.7%), fish (2/195, 1.0%), food animals (1/195, 0.5%), vegetables (1/195, 0.5%), and other sources (4/195, 2.1%). Finnish strains originated from meat (69/197, 35%), FPE (31/197, 15.7%), fish (27/197, 13.7%), birds (25/197, 12.7%), food animals (19/197, 9.6%), raw vegetables (11/197, 5.6%), dairy (8/197, 4.0%), RTE food (3/197, 1.5%), and other sources (4/197, 2.0%).

**Table 2:** **Distribution of the Swiss (CH) and Finnish (FIN) *L. monocytogenes* strains based on serotypes and sources**.

**Source**	**Number of *L. monocytogenes* strains within each serotype**					
	**1/2a**	**1/2b**	**1/2c**	**3a**	**4b**	**Total**
**FOOD-ASSOCIATED ENVIRONMENT**						
CH	9	1	1	0	4	15
FIN	14	8	7	0	2	31
**DAIRY PRODUCTS**						
CH	10	3	0	0	3	16
FIN	7	0	0	1	0	8
**RAW VEGETABLES**						
CH	1	0	0	0	0	1
FIN	4	5	0	1	1	11
**RTE FOODS**						
CH	11	4	0	0	2	17
FIN	2	0	1	0	0	3
**MEAT**						
CH	29	6	10	0	14	59
FIN	54	3	9	0	3	69
**FISH**						
CH	1	1	0	0	0	2
FIN	23	0	0	2	2	27
**FOOD ANIMALS**						
CH	1	0	0	0	0	1
FIN	14	1	0	0	4	19
**BIRDS**						
CH	0	0	0	0	0	0
FIN	13	1	1	0	10	25
**HUMAN LISTERIOSIS**						
CH	41	11	4	0	24	80
FIN	0	0	0	0	0	0
**OTHERS**						
CH	2	0	1	1	0	4
FIN	3	0	0	0	1	4
Total (%)	239 (61%)	44 (11.2%)	34 (8.7%)	5 (1.3%)	70 (17.9%)	392 (100%)

### BC susceptibility of the swiss and finnish *L. monocytogenes* strains

**Figure 1:**
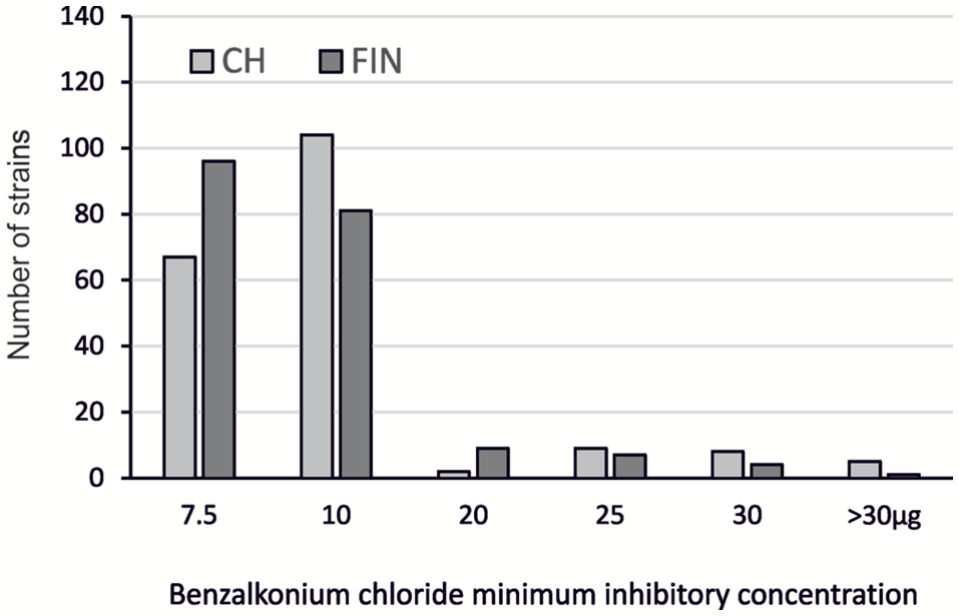
**Distribution of the BC^r^ Swiss (CH) and Finnish (FIN) *L*.* monocytogenes* strains based on BC MICs**.

The BC susceptibility profiles of the Swiss and Finnish *L. monocytogenes* strains were assessed. Benzalkonium chloride MICs ranging from 7.5 to >30 μg ml^−1^ were found (Figure 1; Table 2). Strains with BC MICs ≥20 μg ml^−1^ were classified as BC^r^; strains with BC MICs <20 μg ml^−1^ were classified as BC^s^.

By this definition, the majority (348 out of 392) of strains from both countries were BC^s^. There were however 24 (12.3%) Swiss and 21 (10.6%) Finnish strains classified as BC^r^, with BC MICs ranging from 20 to >30 μg ml^−1^. The largest group within the Swiss strains (104/195; 53.3%) had BC MICs of 10 μg ml^−1^ whereas the largest group within the Finnish strains (96/197; 48.7%) had BC MICs of 7.5 μg ml^−1^.

### Prevalence of BC^r^ strains with respect to isolation sources and serotypes

**Figure 2:**
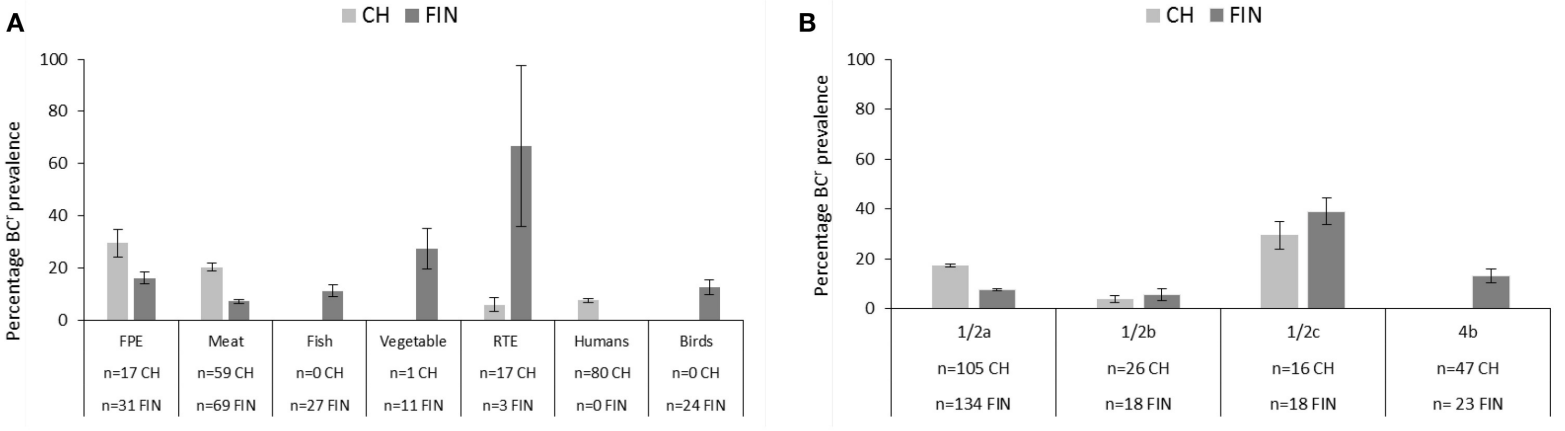
**Bar charts depicting the prevalence (including 95% CIs) and distribution of BC^r^ among Swiss and Finnish *L*.* monocytogenes* strains based on (A)** isolation sources and **(B)** serotypes.

None of the dairy, food animal and other category strains from either Switzerland or Finland from this strain collection exhibited a BC^r^ phenotype. In both countries strains exhibiting BC^r^ phenotypes were recovered from the FPE, raw meat and RTE food categories. Swiss BC^r^ strains also included isolates from human listeriosis cases, whereas the Finnish BC^r^ strains included isolates from raw fish, vegetables, and wild birds. The prevalence of BC^r^ strains in both countries also varied with regard to the isolation sources (Figure 2A).

The overall frequency of a BC^r^ phenotype in the Swiss strains was 12.3% and sources included, in descending order of relative frequency: FPE (5/17, 29.4%), meat (12/59, 20.3%), human isolates (6/80, 7.5%), and RTE food (1/17, 5.9%). The overall frequency of a BC^r^ phenotype in the Finnish strains was 10.2% and the sources included RTE (2/3, 66.7%), vegetables (3/11, 27.3%), the FPE (5/31, 16.1%), fish (3/27, 11.1%), and meat (4/69, 5.8%). Statistical analysis detected significant differences between the prevalence of a BC^r^ phenotype in Swiss vs. Finnish strains that were isolated from meat (CH > FIN) and RTE (CH < FIN) food products (*p* > 0.05). It is possible that such differences might have been biased due to discrepancies in the number of tested strains since there were only three Finnish RTE food strains compared to 17 Swiss strains examined in this category. No significant (*p* > 0.05) differences were detected in BC^r^ prevalence between the FPE, vegetables and raw fish strain categories in the two countries. BC^r^ strain prevalence in human listeriosis (6/80; 7.5%) and bird (3/24; 12.5%) categories in the two countries could however not be compared as they were not represented in both locations.

Prevalence of the BC^r^ strains also varied in each country with regard to the different *L. monocytogenes* serotypes. As expected given the composition of our strain collection, the majority of BC^r^ strains detected in both countries belonged to serotypes 1/2a (28/45, 62.2%) and 1/2c (12/45, 26.7%), although BC^r^ serotype 4b (3/45, 6.7%), and 1/2b (2/45, 4.4%) strains were also found. Interestingly, the highest prevalence of BC resistance was detected in serotype 1/2c strains from both countries (CH 4/16, 25%; FIN 7/18, 38.9%; Figure 2B). The second highest BC^r^ prevalence in Swiss strains was found in serotype 1/2a strains (19/105, 18.1%), and in Finnish strains among serotype 4b strains (3/23, 13%; Figure 2B). Low prevalence of BC resistance was found in serotype 1/2b isolates from both countries (CH: 1/26, 3.8%; FIN: 1/18, 5.5%), and no BC^r^ phenotypes were detected in Swiss serotype 4b (*n* = 47), as well as serotype 3a (*n* = 5) strains from both countries. Statistical comparison revealed significantly higher BC^r^ prevalence among the Swiss (19/105, 18.1% vs. 10/134, 7.5%; *p* < 0.05) serotype 1/2a strains compared to their Finnish counterparts. On the other hand the Swiss serotype 4b strains displayed significantly lower BC^r^ prevalence (0 vs. 13%; *p* < 0.05) compared to those from Finland. No significant differences were observed in BC^r^ prevalence associated with serotype 1/2c and 1/2b strains from the two countries. We are however aware that our observations could be biased due to overall differences in the examined sample sizes between some of the serotype categories in the two countries.

### Genotypes associated with swiss and finnish BC^r^ strains

Molecular genotypes associated with the Swiss and Finnish BC^r^ strains were assessed based on MLST genotyping. The 45 BC^r^ strains from the two countries were assigned to 14 sequence types (ST), which included two newly described sequence types (ST25, ST28; Table 3). BC^r^ strains in both countries belonged predominantly to sequence types ST121 (14/45; 31.1%) and ST9 (11/45; 24.4%) although there were some country specific differences. Sequence type 121 (50 vs. 19%) predominated among the Swiss BC^r^ strains while ST9 (28.6 vs. 20.8%) was predominant in Finnish BC^r^ strains. Sequence types ST403 (*n* = 4), ST204 (*n* = 1), ST25 (*n* = 1), and ST 28 (*n* = 1) were exclusive to Swiss BC^r^ strains whereas ST155 (*n* = 3), ST1 (*n* = 1), ST101 (*n* = 1), ST120 (*n* = 1), ST194 (*n* = 1), and ST515 (*n* = 1) were exclusive to the Finnish BC^r^ strains. Strains were grouped into 10 MLST clonal complexes based on their sequence types showing that BC^r^ in this strain collection is mainly associated with serotype 1/2a, CC121 (16/45; 35.6%) and serotype 1/2c, CC9 (11/45, 24.4%). Overall, most BC^r^ strains belonged to evolutionary genetic lineage II (39/45; 86.7%). There were only five (11.1%) genetic lineage I BC^r^ strains observed, one of which was isolated in Switzerland and four were from Finland. One serotype 1/2c strain that originated from a FPE in Finland was untypable using the current MLST scheme. In this strain, primers for one (*bglA*) out of the seven MLST genes amplified a PCR product bearing a sequence that is unrelated to the *L. monocytogenes bglA* gene.

**Table 3:** **Overview of the Swiss (CH) and Finnish (FIN) BCr strains detected in this study**.

**Strain ID**	**Strain origin**		**Serotype**	**MLST genotypes^a^**			**BC MIC μgml^−1^**		**Efflux pump dependency^b^**	**BCr gene^c^**
	**Country**	**Source**		**CC**	**ST**	**Lineage**	**BC alone**	**BC plus reserpine**		
LM116	FIN	Vegetable	1/2b	CC5	ST5	1	25	30	No	Unknown
N12-2082	CH	Human	1/2a	CC8	ST8	2	25	30	No	Unknown
LT25E	FIN	Vegetable	4b	CC1	ST515	1	30	30	No	Unknown
LL17/3	FIN	Wild bird, feces	4b	CC1	ST1	1	>30	30	Partial	Unknown
LT30E	FIN	Vegetable	1/2a	CC8	ST8	2	25	20	Partial	Unknown
TT107E	FIN	Fish	1/2a	CC155	ST155	2	20	15	Partial	Unknown
N13-0094	CH	Human	1/2a	CC403	ST403	2	25	20	Partial	Unknown
N12-1667	CH	FPE	1/2a	CC403	ST403	2	25	20	Partial	Unknown
N11-1547	CH	Human	1/2a	CC403	ST403	2	25	20	Partial	Unknown
N12-0935	CH	Human	1/2a	CC403	ST403	2	25	20	Partial	Unknown
TT82E	FIN	Fish	1/2a	CC155	ST155	2	25	15	Yes	Unknown
HT45E	FIN	Meat	1/2a	CC155	ST155	2	20	10	Yes	Unknown
LL66/3	FIN	Wild bird, feces	1/2a	CC101	ST101	2	20	7.5	Yes	Unknown
LL1/3	FIN	Wild bird, feces	4b	CC315	ST194	2	20	5	Yes	Unknown
LM84	FIN	RTE food	1/2a	CC8	ST120	2	25	30	No	*emrE*
LK60/1	FIN	Fish	1/2a	CC121	ST121	2	25	30	No	*qacH*
N11-1905	CH	Meat	1/2a	CC121	ST121	2	25	30	No	*qacH*
HL6E	FIN	FPE	1/2c	untypable	untypable	ND	20	20	No	*qacH*
HE152E	FIN	FPE	1/2c	CC9	ST9	2	20	20	No	*qacH*
HT93E/1	FIN	RTE food	1/2c	CC9	ST9	2	20	20	No	*qacH*
HT100E/1	FIN	Meat	1/2c	CC9	ST9	2	20	20	No	*qacH*
L34-s	FIN	Meat	1/2a	CC121	ST121	2	30	30	No	*qacH*
MJL14	FIN	FPE	1/2a	CC121	ST121	2	30	30	No	*qacH*
HT65E/1	FIN	Meat	1/2a	CC121	ST121	2	30	30	No	*qacH*
N13-0119	CH	Human	1/2a	CC121	ST121	2	30	30	No	*qacH*
N12-0367	CH	Human	1/2a	CC121	ST121	2	30	30	No	*qacH*
Lm 760	CH	Meat	1/2c	CC9	ST9	2	20	20	No	*qacH*
N11-2543	CH	FPE	1/2a	CC121	ST121	2	30	30	No	*qacH*
N12-0571	CH	Meat	1/2a	CC121	ST121	2	30	30	No	*qacH*
Lm S1	CH	FPE	1/2a	CC121	ST121	2	30	30	No	*qacH*
N11-1218	CH	Meat	1/2a	CC121	ST25	2	>30	30	Partial	*qacH*
HT69E	FIN	Meat	1/2c	CC9	ST9	2	20	15	Partial	*qacH*
HE28E	FIN	FPE	1/2c	CC9	ST9	2	25	20	Partial	*qacH*
Lm 217	CH	Meat	1/2a	CC9	ST9	2	25	20	Partial	*qacH*
Lm 25/9	CH	Meat	1/2c	CC9	ST9	2	20	15	Partial	*qacH*
Lm 89	FIN	FPE	1/2c	CC9	ST9	2	25	20	Partial	*qacH*
N12-0494	CH	Meat	1/2a	CC121	ST121	2	25	20	Partial	*qacH*
N12-2229	CH	RTE food	1/2a	CC121	ST121	2	25	30	No	*brcABC*
N12-0644	CH	Meat	1/2c	CC9	ST9	2	>30	>30	No	*brcABC*
N12-2271	CH	Meat	1/2c	CC9	ST9	2	>30	>30	No	*brcABC*
N12-2118	CH	Meat	1/2a	CC121	ST121	2	30	30	No	*brcABC*
N13-0288	CH	Meat	1/2a	CC121	ST28	2	30	30	No	*brcABC*
N13-0369	CH	Meat	1/2a	CC121	ST121	2	30	30	No	*brcABC*
Lm S9	CH	FPE	1/2a	CC204	ST204	2	>30	>30	No	*brcABC*
Lm S2	CH	FPE	1/2b	CC5	ST5	1	>30	30	No	*brcABC*

a *MLST types and genetic lineages were assigned based on the L. monocytogenes MLST database website (http://bigsdb.web.pasteur.fr)*.

b *Efflux pump dependency: yes: BC MIC decreases by ≥10 μg ml^−1^ in the presence of the efflux pump inhibitor reserpine. Partial: BC MIC decreases by <10 μg ml^−1^ in presence of reserpine. No: BC MIC was not affected by the presence of reserpine*.

c *BC^r^ gene presence determined by PCR*.

### Prevalence of BC resistance genes in swiss and finnish BC^r^ strains

Benzalkonium chloride resistant strains were also examined for the distribution of genes encoding the three efflux pump systems (*brcABC, qacH*, and *emrE*) currently known to confer BC resistance in *L. monocytogenes* (Table 3). The PCR-based analysis detected genes associated with such efflux pump systems in 79% (19/24) Swiss and 57% (12/21) Finnish BC^r^ strains, respectively. Swiss strains harbored both *qacH* (11/24; 45.8%) and *brcABC* (8/24, 33.3%) associated genes, and no strains harboring *emrE* were found. A majority of the BC^r^ strains from Finland harbored *qacH* genes (11/21; 52.4%), no *bcrABC* genes were found, and in one BC^r^ strain, an *emrE* gene was detected. With respect to associated serotypes and MLST genotypes, the *qacH* genes were detected in serotype 1/2a, CC121 (ST121 and ST28), serotype 1/2a, CC204 (ST204), and serotype 1/2c, CC9 (ST9) strains. The *brcABC* genes were associated with serotype 1/2a, CC121 (ST121 and ST25), serotype 1/2b, CC5, and serotype 1/2c, CC9 strains. The *emrE* gene was associated with a serotype 1/2a, CC8 (ST120) strain. In terms of origins, the *qacH*-encoding strains came from FPE, raw meat, fish RTE food, and human listeriosis cases. The *brcABC* harboring strains were from FPE and raw meat, and the *emrE* strain originated from an RTE food product. None of the three known BC resistance determinants were detected in 21% (5/24) and 43% (9/21) of the Swiss and Finnish BC^r^ strains, respectively (Table 3). This group included serotype 4b, CC1 (ST1 and ST515), serotype 1/2a, CC8 (ST8), CC101 (ST101), CC155 (ST155), and serotype 1/2b, CC5 (ST5) strains, which were isolated from diverse sources. At this stage, sequence alterations affecting PCR primer binding sites across different strains cannot be completely ruled out as a possible reason for false negative results in some of the BC^r^ strains found to lack the known BC^r^ genes.

### Role of efflux pump activity in swiss and finnish BC^r^ strains

A screen with reserpine showed that the BC resistance in 4 out of 45 strains depended on reserpine sensitive efflux pump systems. The BC resistance in an additional 14 strains was classified as partially efflux pump dependent while the addition of reserpine had no effect on the BC MIC in 27 strains. As mentioned above, a subset of 14 BC^r^ strains lacked known BC resistance genes. Reserpine addition had no impact on BC MICs in four of those strains. The BC resistance in an additional three strains was classified as efflux pump dependent, and in seven strains as partially efflux dependent. (Table 3).

## Discussion

In this study 392 *L. monocytogenes* strains recovered from human clinical listeriosis, food products and production environments, food animals, and wild birds in Switzerland and Finland were analyzed with respect to BC resistance. The strain collections could not be exactly matched or balanced with respect to origin, due to country specific differences in the type of food typically produced and limited availability of isolates. Although it remains unclear how well the strain collection represents the true distribution of strains in these two geographical locations, the large number of strains included in this study is likely to balance some of the potential bias. The vast majority of analyzed strains belonged to serotype 1/2a, 1/2b, 1/2c, and 4b, which are typical *L. monocytogenes* serotypes found in food, the food processing environment and human clinical cases (Orsi et al., 2011).

The prevalence of BC^r^ strains of 11.4% amongst our strains is at the lower end of what other authors have found. In comparison, prevalences of BC-resistant strains determined in other studies range from ~10% in strains isolated from fish and poultry factories (Aase et al., 2000), human clinical cases and food (Mereghetti et al., 2000; Ratani et al., 2012); 18–26% in strains isolated from food in Switzerland (Ebner et al., 2015) and China (Xu et al., 2014; Jiang et al., 2016) to 61% in strains originating from fish processing (Soumet et al., 2005) and the human clinical, food, and food production environment (Mullapudi et al., 2008; Dutta et al., 2013). These differences are partially due to the different methods used as well as differences in the definition of resistance across studies. The range of cutoffs for BC-resistance from 4 to 16 μg ml^−1^ in these studies (Aase et al., 2000; Mereghetti et al., 2000; Soumet et al., 2005; Mullapudi et al., 2008; Dutta et al., 2013; Xu et al., 2014; Ebner et al., 2015; Jiang et al., 2016) is a consequence of the commonly used method to determine BC-resistance relative to the MIC that inhibits a majority of strains. Working concentrations of BC in commercial products used in the food processing environment typically range from 500 to 1,000 μg ml^−1^ (Hegstad et al., 2010). However, *L. monocytogenes* preferably survives in niches with low accessibility for cleaning where the actual concentration of disinfectants is hard to predict. Defining relative cutoffs for BC-resistance is therefore a reasonable approach to focus on the strains that are most likely to have a selective advantage during repeated disinfection procedures.

There was no clear correlation between resistance to BC and country of origin. In our study, serotype 1/2a comprised the largest number of BC^r^ strains although the relative prevalence of BC^r^ was highest among the serotype 1/2c strains. Other authors have found varying fractions of BC^r^ serotype 1/2a, 1/2b, 1/2c, and 4b strains of *L. monocytogenes* (ranging from 7 to 60% for serotype 1/2a; from 0 to 51% for serotype 1/2b; from 22 to 75% for serotype 1/2c and from 0 to 100% for serotype 4b; Mereghetti et al., 2000; Romanova et al., 2002; Soumet et al., 2005; Mullapudi et al., 2008; Ratani et al., 2012; Xu et al., 2014; Jiang et al., 2016). This wide range is to be expected, given the often relatively small sample sizes and the low discriminatory power of serotyping (Datta et al., 2013).

In contrast, analysis by MLST revealed that CC121 and CC9 are overrepresented among BC^r^ strains carrying *brcABC* and *qacH* genes, which confirms the results of an earlier Swiss study (Ebner et al., 2015). Both of these clonal complexes are commonly found worldwide in association with food and clinical cases (Chenal-Francisque et al., 2011). A large study analyzing the population biology of 1696 strains of *L. monocytogenes* by core genome MLST indicated a broad range of strains carrying *brcABC* and *qacH* genes (including a cluster of CC121 strains) while *emrE* seems to be limited to sublineage 8 strains (comprising CC8, ST120; Moura et al., 2016). Incidentally, the only strain in our panel carrying the *emrE* gene also belongs to CC8, ST120. None of the BC^r^ strains lacking *brcABC, emrE*, and *qacH* belonged to CC121 or CC9. Instead, these strains belong to a more diverse set of seven sequence types including a cluster of four CC403 strains, a clonal complex that seems to be relatively rare and largely found in Europe with only five entries in the MLST database of the Institute Pasteur (http://bigsdb.web.pasteur.fr).

Our dataset provides several lines of evidence for mechanisms of BC resistance other than the known efflux pumps that may work either alone or in conjunction with the products of the *bcrABC, qacH*, and *emrE* genes. (i) Fourteen BC^r^ strains carried none of the known BC efflux pumps as determined by PCR. (ii) In eleven of these strains, reserpine screening indicated that efflux pumps other than those coded by *emrE, bcrABC*, and *qacH* play at least a partial role in conferring resistance to BC. (iii) Out of the 31 BC^r^ strains carrying genes encoding for known efflux pumps, 24 showed no reduction of the BC MIC in the presence of reserpine. This may indicate the presence of additional, yet unknown genes that confer BC resistance via a mechanism other than efflux pumps in these strains. Alternatively, reserpine may not be equally effective against all efflux pumps. In fact, other authors (Ortiz et al., 2015) found no difference in BC MIC after the addition of reserpine in a strain carrying the Tn6188 transposon (coding for *qacH* Müller et al., 2013), and a study analyzing efflux pumps conferring multidrug resistance to *Staphylococcus aureus* showed that reserpine failed to identify their presence in a considerable number (72/128, 61%) of strains (Frempong-Manso et al., 2009). While the addition of reserpine might not be a reliable method to exclude the presence of efflux pumps, in instances where it does exert an effect on the MIC of a given antimicrobial the presence of efflux pumps can be assumed (Godreuil et al., 2003; Soumet et al., 2005; Romanova et al., 2006; Xu et al., 2014).

Further, analysis of the BC^r^ strains in our panel that do not code for *bcrABC, qacH*, and *emrE* genes might help identify these additional factors involved in BC resistance. For instance, increased transcription of the multidrug resistance transporter *lde* has been reported in response to BC (Rakic-Martinez et al., 2011). Other than the activity of efflux pumps, modifications of the cell wall may potentially increase tolerance of BC by *L. monocytogenes* (McDonnell and Russell, 1999). This is supported by evidence from several studies: Mereghetti et al. (2000) observed an association between BC resistance and failure of phage-based subtyping methods, which may indicate modifications in the wall teichoic acids. In addition, transcriptional analysis of the response to QAC revealed upregulation of peptidoglycan synthesis pathways (Fox et al., 2011), and To et al. (2002) found a shift in fatty acid composition in one BC-adapted strain compared to the parent strain.

In conclusion, BC^r^ strains of *L. monocytogenes* are present and should be monitored in the Swiss and Finnish food production environment with a special focus on strains that belong to CC9 and CC121. One strain from Finland carried the *emrE* gene, which to our knowledge is the first time the *emrE* gene has been described in a strain of *L. monocytogenes* originating outside of Canada. Additionally, we found BC resistance in strains lacking all of the known BC resistance genes, indicating the presence of yet unknown mechanisms of BC resistance.

## Author contributions

TT and HK designed and supervised the study. ABM, AM, and AP performed the experiments. ABM, TT, HK, and CG analyzed the data and wrote the manuscript.

## Funding

ABM was partly funded by the University of Zurich.

### Conflict of interest statement

The authors declare that the research was conducted in the absence of any commercial or financial relationships that could be construed as a potential conflict of interest.

## References

[B1] Aase B. Sundheim G. Langsrud S. Rørvik L. M. (2000). Occurrence of and a possible mechanism for resistance to a quaternary ammonium compound in *Listeria monocytogenes*. Int. J. Food Microbiol. 62, 57–63. 10.1016/S0168-1605(00)00357-3 11139022

[B2] Bergholz T. M. den Bakker H. C. Fortes E. D. Boor K. J. Wiedmann M. (2010). Salt stress phenotypes in *Listeria monocytogenes* vary by genetic lineage and temperature. Foodborne. Pathog. Dis. 7, 1537–1549. 10.1089/fpd.2010.0624 20707723 PMC3022828

[B3] Chenal-Francisque V. Lopez J. Cantinelli T. Caro V. Tran C. Leclercq A.. (2011). Worldwide distribution of major clones of *Listeria monocytogenes*. Emerg. Infect. Dis. 17, 1110–1112. 10.3201/eid/1706.101778 21749783 PMC3358213

[B4] Crim S. M. Griffin P. M. Tauxe R. Marder E. P. Gilliss D. Cronquist A. B.. (2015). Preliminary incidence and trends of infection with pathogens transmitted commonly through food - Foodborne diseases active surveillance network, 10 U.S. sites, 2006-2014. MMWR. Morb. Mortal. Wkly. Rep. 64, 495–499. 25974634 PMC4584825

[B5] Datta A. R. Laksanalamai P. Solomotis M. (2013). Recent developments in molecular sub-typing of *Listeria monocytogenes*. Food Addit. Contam. Part A Chem. Anal. Control Expo. Risk Assess. 30, 1437–1445. 10.1080/19440049.2012.728722 23061558

[B6] de Valk H. Jacquet C. Goulet V. Vaillant V. Perra A. Simon F.. (2005). Surveillance of Listeria infections in Europe. Eur. Surveill. 10, 251–255. Available online at: http://www.eurosurveillance.org/images/dynamic/EQ/v05n04/v05n04.pdf 16282642

[B7] Dutta V. Elhanafi D. Kathariou S. (2013). Conservation and distribution of the benzalkonium chloride resistance cassette bcrABC in *Listeria monocytogenes*. Appl. Environ. Microbiol. 79, 6067–6074. 10.1128/AEM.01751-13 23892748 PMC3811391

[B8] Ebner R. Stephan R. Althaus D. Brisse S. Maury M. Tasara T. (2015). Phenotypic and genotypic characteristics of *Listeria monocytogenes* strains isolated during 2011–2014 from different food matrices in Switzerland. Food Control 57, 321–326. 10.1016/j.foodcont.2015.04.030

[B9] Elhanafi D. Dutta V. Kathariou S. (2010). Genetic characterization of plasmid-associated benzalkonium chloride resistance determinants in a *Listeria monocytogenes* strain from the 1998-1999 outbreak. Appl. Environ. Microbiol. 76, 8231–8238. 10.1128/AEM.02056-10 20971860 PMC3008257

[B10] Ferreira V. Wiedmann M. Teixeira P. Stasiewicz M. J. (2014). *Listeria monocytogenes* persistence in food-associated environments: epidemiology, strain characteristics, and implications for public health. J. Food Prot. 77, 150–170. 10.4315/0362-028X.JFP-13-150 24406014

[B11] Fox E. M. Leonard N. Jordan K. (2011). Physiological and transcriptional characterization of persistent and non-persistent *Listeria monocytogenes* isolates. Appl. Environ. Microbiol. 77, 6559–6569. 10.1128/AEM.05529-11 21764947 PMC3187160

[B12] Frempong-Manso E. Raygada J. L. DeMarco C. E. Seo S. M. Kaatz G. W. (2009). Inability of a reserpine-based screen to identify strains overexpressing efflux pump genes in clinical isolates of Staphylococcus aureus. Int. J. Antimicrob. Agents 33, 360–363. 10.1016/j.ijantimicag.2008.10.016 19097759

[B13] Gilmoure M. W. Graham M. Van Domselaar G. Tyler S. Kent H. Trout-Yakel K. M. (2010). High-throughput genome sequencing of two *Listeria monocytogenes* clinical isolates during a large foodborne outbreak. BMC.Genomics 11:120 10.1186/1471-2164-11-120 20167121 PMC2834635

[B14] Godreuil S. Galimand M. Gerbaud G. Jacquet C. Courvalin P. (2003). Efflux pump Lde is associated with fluoroquinolone resistance in *Listeria monocytogenes*. Antimicrob. Agents Chemother. 47, 704–708. 10.1128/AAC.47.2.704-708.2003 12543681 PMC151722

[B15] Hegstad K. Langsrud S. Lunestad B. T. Scheie A. A. Sunde M. Yazdankhah S. P. (2010). Does the wide use of quaternary ammonium compounds enhance the selection and spread of antimicrobial resistance and thus threaten our health? Microb. Drug Resist. 16, 91–104. 10.1089/mdr.2009.0120 20370507

[B16] Jiang X. Yu T. Liang Y. Ji S. Guo X. Ma J.. (2016). Efflux pump-mediated benzalkonium chloride resistance in *Listeria monocytogenes* isolated from retail food. Int. J. Food Microbiol. 217, 141–145. 10.1016/j.ijfoodmicro.2015.10.022 26513255

[B17] Kovacevic J. Mesak L. R. Allen K. J. (2012). Occurrence and characterization of *Listeria* spp. in ready-to-eat retail foods from Vancouver, British Columbia. Food Microbiol. 30, 372–378. 10.1016/j.fm.2011.12.015 22365350

[B18] Kovacevic J. Ziegler J. Walecka-Zacharska E. Reimer A. Kitts D. D. Gilmour M. W. (2015). Tolerance of *Listeria monocytogenes* to quaternary ammonium sanitizers is mediated by a novel efflux pump encoded by emrE. Appl. Environ. Microbiol. 82, 939–953. 10.1128/AEM.03741-15 26590290 PMC4725271

[B19] Langsrud S. Sidhu M. S. Heir E. Holck A. (2003). Bacterial disinfectant resistance—a challenge for the food industry. Int. Biodeterior. Biodegradation 51, 283–290. 10.1016/S0964-8305(03)00039-8

[B20] Maertens de Noordhout C. Devleesschauwer B. Angulo F. J. Verbeke G. Haagsma J. Kirk M.. (2014). The global burden of listeriosis: a systematic review and meta-analysis. Lancet Infect. Dis. 14, 1073–1082. 10.1016/S1473-3099(14)70870-9 25241232 PMC4369580

[B21] McDonnell G. Russell A. D. (1999). Antiseptics and disinfectants: activity, action, and resistance. Clin. Microbiol. Rev. 12, 147–179. 9880479 10.1128/cmr.12.1.147 PMC88911

[B22] Mereghetti L. Quentin R. Marquet-Van Der Mee N. Audurier A. (2000). Low sensitivity of *Listeria monocytogenes* to quaternary ammonium compounds. Appl. Environ. Microbiol. 66, 5083–5086. 10.1128/AEM.66.11.5083-5086.2000 11055967 PMC92423

[B23] Moura A. Criscuolo A. Pouseele H. Maury M. M. Leclercq A. Tarr C.. (2016). Whole genome-based population biology and epidemiological surveillance of *Listeria monocytogenes*. Nat. Microbiol. 2:16185. 10.1038/nmicrobiol.2016.185 27723724 PMC8903085

[B24] Mullapudi S. Siletzky R. M. Kathariou S. (2008). Heavy-metal and benzalkonium chloride resistance of *Listeria monocytogenes* isolates from the environment of turkey-processing plants. Appl. Environ. Microbiol. 74, 1464–1468. 10.1128/AEM.02426-07 18192428 PMC2258618

[B25] Müller A. Rychli K. Muhterem-Uyar M. Zaiser A. Stessl B. Guinane C. M.. (2013). Tn6188 - a novel transposon in *Listeria monocytogenes* responsible for tolerance to benzalkonium chloride. PLoS ONE 8:e76835. 10.1371/journal.pone.0076835 24098567 PMC3788773

[B26] Orsi R. H. den Bakker H. C. Wiedmann M. (2011). *Listeria monocytogenes* lineages: Genomics, evolution, ecology, and phenotypic characteristics. Int. J. Med. Microbiol. 301, 79–96. 10.1016/j.ijmm.2010.05.002 20708964

[B27] Ortiz S. Lopez-Alonso V. Rodriguez P. Martinez-Suarez J. V. (2015). The connection between persistent, disinfectant-resistant *Listeria monocytogenes* strains from two geographically separate iberian pork processing plants: evidence from comparative genome analysis. Appl. Environ. Microbiol. 82, 308–317. 10.1128/AEM.02824-15 26497458 PMC4702620

[B28] Popovic I. Heron B. Covacin C. (2014). Listeria: an Australian perspective (2001-2010). Foodborne. Pathog. Dis. 11, 425–432. 10.1089/fpd.2013.1697 24697613

[B29] Ragon M. Wirth T. Hollandt F. Lavenir R. Lecuit M. Le M.. (2008). A new perspective on *Listeria monocytogenes* evolution. PLoS Pathog. 4:e1000146. 10.1371/journal.ppat.1000146 18773117 PMC2518857

[B30] Rakic-Martinez M. Drevets D. A. Dutta V. Katic V. Kathariou S. (2011). *Listeria monocytogenes* strains selected on ciprofloxacin or the disinfectant benzalkonium chloride exhibit reduced susceptibility to ciprofloxacin, gentamicin, benzalkonium chloride, and other toxic compounds. Appl. Environ. Microbiol. 77, 8714–8721. 10.1128/AEM.05941-11 22003016 PMC3233111

[B31] Ratani S. S. Siletzky R. M. Dutta V. Yildirim S. Osborne J. A. Lin W.. (2012). Heavy metal and disinfectant resistance of *Listeria monocytogenes* from foods and food processing plants. Appl. Environ. Microbiol. 78, 6938–6945. 10.1128/AEM.01553-12 22843526 PMC3457492

[B32] Romanova N. A. Wolffs P. F. Brovko L. Y. Griffiths M. W. (2006). Role of efflux pumps in adaptation and resistance of *Listeria monocytogenes* to benzalkonium chloride. Appl. Environ. Microbiol. 72, 3498–3503. 10.1128/AEM.72.5.3498-3503.2006 16672496 PMC1472371

[B33] Romanova N. Favrin S. Griffiths M. W. (2002). Sensitivity of *Listeria monocytogenes* to sanitizers used in the meat processing industry. Appl. Environ. Microbiol. 68, 6405–6409. 10.1128/AEM.68.12.6405-6409.2002 12450868 PMC134375

[B34] Smith J. L. Liu Y. Paoli G. C. (2013). How does *Listeria monocytogenes* combat acid conditions? Can. J. Microbiol. 59, 141–152. 10.1139/cjm-2012-0392 23540331

[B35] Soumet C. Ragimbeau C. Maris P. (2005). Screening of benzalkonium chloride resistance in *Listeria monocytogenes* strains isolated during cold smoked fish production. Lett. Appl. Microbiol. 41, 291–296. 10.1111/j.1472-765X.2005.01763.x 16108923

[B36] To M. S. Favrin S. Romanova N. Griffiths M. W. (2002). Postadaptational resistance to benzalkonium chloride and subsequent physicochemical modifications of *Listeria monocytogenes*. Appl. Environ. Microbiol. 68, 5258–5264. 10.1128/AEM.68.11.5258-5264.2002 12406712 PMC129876

[B37] Walker S. J. Archer P. Banks J. G. (1990). Growth of *Listeria monocytogenes* at refrigeration temperatures. J. Appl. Bacteriol. 68, 157–162. 10.1111/j.1365-2672.1990.tb02561.x 2108109

[B38] Xu D. Li Y. Zahid M. S. Yamasaki S. Shi L. Li J. R.. (2014). Benzalkonium chloride and heavy-metal tolerance in *Listeria monocytogenes* from retail foods. Int. J. Food Microbiol. 190, 24–30. 10.1016/j.ijfoodmicro.2014.08.017 25173916

